# Investigating the Cortical Correlates of Singing: Potential Neural Benefits of Choir for Persons With Dementia

**DOI:** 10.1093/geroni/igab046.498

**Published:** 2021-12-17

**Authors:** Nicholas Tamburri, Debra Sheets, Drew Halliday, Andre Smith, Stuart MacDonald

**Affiliations:** University of Victoria, Victoria, British Columbia, Canada

## Abstract

Through leveraging the known advantages of musical engagement and socialization, choir interventions are known to facilitate psychological and cognitive benefits for persons with dementia (PwD). Surprisingly, no research has explored whether social singing may also confer neurological advantages. In this study, we employed functional near infrared spectroscopy (fNIRS) to investigate the cortical correlates of both social and solo singing in PwD (n=13). Paired-sample t-tests were used to evaluate within-person differences in frontal cortical activation between the social vs solo singing. Results showed significant activation differences in three frontal channels, with social singing requiring comparatively less frontocortical activation. These findings indicate potential neural benefits of social singing – with less frontal activation being a proxy for greater reliance on intact proceduralized systems – and serve to highlight the utility of fNIRS in better understanding the neural correlates underlying the benefits of social singing interventions for PwD.

